# Spermidine supplementation in honey bees: Autophagy and epigenetic modifications

**DOI:** 10.1371/journal.pone.0306430

**Published:** 2024-07-01

**Authors:** Danijela Kojić, Jelena Spremo, Srđana Đorđievski, Tatjana Čelić, Elvira Vukašinović, Ivan Pihler, Jelena Purać

**Affiliations:** 1 Faculty of Science, Department of Biology and Ecology, University of Novi Sad, Novi Sad, Serbia; 2 Faculty of Agriculture, Department of Animal Sciences, University of Novi Sad, Novi Sad, Serbia; University of the Pacific, UNITED STATES

## Abstract

Polyamines (PAs), including putrescine (Put), spermidine (Spd), and spermine (Spm), are essential polycations with wide-ranging roles in cellular functions. PA levels decline with age, making exogenous PA supplementation, particularly Spd, an intriguing prospect. Previous research in honey bees demonstrated that millimolar Spd added to their diet increased lifespan and reinforced oxidative resilience. The present study is aimed to assess the anti-aging effects of spermidine supplementation at concentrations of 0.1 and 1 mM in honey bees, focusing on autophagy and associated epigenetic changes. Results showed a more pronounced effect at the lower Spd concentration, primarily in the abdomen. Spd induced site-specific histone 3 hypoacetylation at sites K18 and 27, hyperacetylation at K9, with no change at K14 in the entire body. Additionally, autophagy-related genes (ATG3, 5, 9, 13) and genes associated with epigenetic changes (*HDAC1*, *HDAC3*, *SIRT1*, *KAT2A*, *KAT6B*, *P300*, *DNMT1A*, *DNMT1B*) were upregulated in the abdomens of honey bees. In conclusion, our findings highlight profound epigenetic changes and autophagy promotion due to spermidine supplementation, contributing to increased honey bee longevity. Further research is needed to fully understand the precise mechanisms and the interplay between epigenetic alterations and autophagy in honey bees, underscoring the significance of autophagy as a geroprotective mechanism.

## Introduction

Polyamines (PAs), such as putrescine (Put), spermidine (Spd), and spermine (Spm), are low molecular weight polycations that are ubiquitous and essential in all living systems. PAs participate in numerous biological processes. They are important for cell growth, differentiation, and stress protection, having a role in aging, memory performance, metabolic disorders, and diseases [1–4]. As cellular polycations, PAs interact with negatively charged molecules such as DNA, RNA, and proteins. By changing the structure and stability of nucleic acids and proteins, PAs can change enzyme activity, remodel chromatin, promote binding between proteins and DNA, and regulate gene expression by modulating the rate of transcription and ribosomal-mediated translation (reviewed in [5]). Since intracellular PA concentration is critical for all these functions, the cellular pool of PAs is tightly regulated by their biosynthesis, degradation, and transport via unusual and conserved regulatory features (reviewed in [6]). Interestingly, PAs content declines with aging, but exogenous supplies of PAs, particularly Spd, can replenish cellular PAs with beneficial effects on lifespan and health promotion in model organisms and humans [7–9] (reviewed in [10]). Spermidine has been the most studied polyamine in terms of its anti-aging effect and has recently been proposed as a longevity elixir. Spd administration extended the lifespan of yeast, flies, worms, and human immune cells, while reducing age-related oxidative damage in aging mice [9,11].

Autophagy is considered an essential mechanism for the anti-aging activity of Spd [9–12]. Moreover, the beneficial anti-aging effect of Spd is accompanied by the induction of signs of autophagy in tested model organisms [9–11]. Autophagy is the main mechanism of recycling intracellular components (usually malfunctioning and damaged proteins, cellular debris, or whole organelles) within lysosomes (reviewed in [13]). There is a complex network of autophagy regulation, but all these pathways have an effect on autophagy-related (ATG) proteins, changing their post-translational modifications or altering gene expression of these proteins (reviewed in [14]). Autophagy declines with age in various organisms and has been linked to many age-related diseases (reviewed in [15]).

There is a lack of information about cellular events linked to high Spd levels and autophagy induction. Generally, Spd alters the epigenetic landscape, which may have an impact on specific promoters and gene regions that control the transcription of genes linked with autophagy [9,10,12]. Global histone deacetylation induced by Spd administration was observed, which was ascribed to specific inhibition of histone acetyltransferases (KATs or formerly HATs) [9,12,16]. The open question is whether Spd can trigger autophagy via post-translational mechanisms that alter the acetylation of autophagic non-histone cytosolic proteins through KAT inhibition, considering that many of them are KAT substrates, including some ATG proteins and transcription factors [12,17]. Additionally, Spd, by stimulating the translocation of some histone deacetylases (HDACs) to the nucleus and preventing deacetylation of target cytosolic proteins, could promote autophagy flux as well [18]. Soda et al. [19] demonstrated that exogenous PAs increased DNA methyltransferase (DNMT) activity and reduced age-associate global alternation in DNA methylation status in old mice, suggesting that Spd may be responsible for additional epigenetic changes.

It has been shown before that Spd supplementation improved honey bee survival and average lifespan and influenced endogenous PA levels [20]. Additionally, exogenous Spd improved oxidative status and increased vitellogenin gene expression, indicating that antioxidative activity could be one of the geroprotective mechanisms of Spd in honey bees. Honey bees are an interesting organism for this type of research for at least two reasons. First, honey bees are commonly used as model organisms in aging studies because of their extreme phenotypic plasticity with regard to lifespan among closely related siblings [21,22]. Second, honey bees are one of the most important global pollinators, with a significant impact on biodiversity and the economy, yet there has been an increasing loss of managed honey bee colonies over the last few decades [23].

In this study, the objective was to investigate whether spermidine exerts its anti-aging effects in bees through the process of autophagy and the associated epigenetic changes. To address this question, the relative expression of selected genes associated with autophagy (*ATG* genes), histone acetylation (*HDACs* and *KATs* genes), and DNA methylation (*DNMT* genes) was assessed in honey bees supplemented with Spd. In addition, four epigenetic modifications of histone H3 using Western blot were analyzed.

## Materials and methods

### Honey bees collection and formation of experimental groups

The experiment was conducted in June 2022. In this study, all worker honey bees originated from a single hive from the apiary located at the Fruška Gora mountain (45°22’ N; 19°53’ E), near Novi Sad, Serbia. The hive was maintained by an experienced beekeeper according to good beekeeping practice [24]. The bee colony was strong (approximately 20000–30000 bees), with no clinical signs of infectious diseases. Before the experiment set-up, two frames with ready-to-emerge worker brood were collected from the apiary and placed in an incubator at 34°C and 65% humidity. After 24h brood frames were replaced with honey and bee bread frames to provide food for newly emerged honey bees, and the temperature was lowered to 28°C. After 5 days, young worker honey bees were transferred into 2 L plastic boxes, each containing approximately 30 bees. Three experimental groups were formed: control (C) and two supplemented groups (S_0.1_ and S_1_). In the control groups, honey bees were fed sucrose solution (50% w/v), while the experimental groups S_0.1_ and S_1_ were fed sucrose solution supplemented with 0.1 mM and 1 mM spermidine, respectively. These concentrations were selected based on a previous study of the supplementation of honey bees with a wide range of spermidine concentrations [20], as those that showed a positive effect on the survival and lifespan of honey bees. Each group included three biological replicates. Food was prepared and changed daily, and dead bees were removed. After 17 days, the bees were collected in a plastic vial, and immediately frozen with dry ice, and stored at -80°C for further analysis. According to the aforementioned study [20], the selected experiment’s duration is also a time point when spermidine supplementation has a significant impact on the physiology of honey bees.

### Total RNA isolation and cDNA synthesis

For total RNA isolation from honey bees, body segments (head, thorax, and abdomen) were separated. For each biological replicate, three heads or abdomens were pooled and homogenized in a microtube with pestle with the addition of RNA Extracol (EurX). Chloroform was added and the homogenate was allowed to separate into a clear upper aqueous layer, an interphase, and a lower organic layer. RNA remains in the aqueous phase, which was transferred to clear microtube and RNA was precipitated with isopropanol. RNA precipitate was diluted in 30–50 μl of RNase/DNase-free water (Sigma-Aldrich). RNA concentration was measured at 260 nm using a BioSpec-nano spectrophotometer (Shimatzu), and the purity of total RNA was determined as the 260/280 absorbance ratio. After the estimation of purity and concentration, total RNA was diluted to 500 ng/μl and stored at −80°C. The synthesis of cDNA was carried out using the QuantiTect Reverse Transcription Kit (Qiagen) according to the manufacturer’s protocol, starting with 1 μg of total RNA. In this kit, genomic DNA in RNA samples was removed using the gDNA Wipeout Buffer and reverse transcription was performed using Quantiscript Reverse Transcriptase, RT Buffer and RT Primer Mix optimized for high cDNA yield for all regions of the RNA transcripts. After reverse transcription, the obtained samples were stored at -20°C.

### Real-time PCR

Real-time PCR was used to measure the relative expression of selected genes associated with autophagy (*ATG3*, *5*, *9*, *13*), histone deacetylases (*HDAC1*, *HDAC3*, *SIRT1*), histone acetyltransferases (*P300*, *KAT6B*, *KAT2A*), and DNA methyltransferases (*DNMT1A*, *DNMT1B*, *DNMT3*). Data about primers are listed in S1 Table. Genes for β-actin (*ActB*) and ribosomal protein 49 (*Rp49*) were used as an endogenous control. The suitability of these genes, as an endogenous control in relative gene expression analysis, has previously been confirmed [25]. PCR reaction mix consisted of 7 μl of 2×SYBR Green PCR Master Mix (Applied Biosystems), 500 nM of each primer, and 40 ng cDNA in a total volume of 14 μl. Quantitative PCR on the cDNA products was carried out using MasterCycler RealPlex4 (Eppendorf). Amplification program included initial preincubation at 95°C (10 min) and 40 cycles of 95°C (15 sec) and 60°C (1 min), with an additional step for melting curve analysis to confirm amplification of a single gene product.

### Western blot, quantification of histone acetylation

For Western blot analysis, histones were extracted from whole bees (three bees from each biological replicate) using the Histone Extraction Kit (Abcam ab113476) according to the manufacturer’s instructions. The concentration of total protein in extracts was quantified by Bradford method [26], using BSA as a standard. Histone extracts were separated by SDS-PAGE on 15% gel [27] with an equal amount of loaded proteins (30 μg/well), and then transferred to a nitrocellulose membrane (2 μm, Bio-Rad) at constant voltage (100V) for 70 min. Nonspecific binding sites were blocked by incubating membranes in 5% non-fat dry milk in TBST (20 mM TRIS, 150 mM NaCl, 0.1% Tween) for 1 h at room temperature. The membranes were then incubated overnight at 4°C with primary antibodies against total histone H3, which served as a loading control, or histone H3 acetylated at specific lysine (H3K27ac, H3K9ac, H3K18ac, H3K14ac), followed by incubation with a second goat anti-rabbit antibody conjugated with horseradish peroxidase for 1 h at room temperature. The specifications and dilution ratios of the antibodies used are listed in S2 Table. The signal was detected using an ultra-sensitive enhanced chemiluminescent substrate (SuperSignal™ West Femto Maximum Sensitivity Substrate) by myECL Imager (Thermo Scientific). The intensity of protein bands was measured using ImageJ software [28], and normalized to the band intensity of H3 as a loading control on the same blot. The acetylation of each sample was relativized according to the acetylation rates of control samples (considered as 1) that were on the same blot.

### Statistical analysis

Relative gene expression was analysed by REST 2009 software (Qiagen). REST2009 software uses adapted version of delta-delta Ct method, published by Pfaffl (2001) [29], which takes into account the different PCR efficiency of the gene of interest and reference genes.The relative difference between the control and each experimental group was calculated and tested for statistical significance by the integrated Bootstrap randomization test (2000 iterations) for p<0.05 confidence intervals.

Western blot data were analysed with a one-way ANOVA followed by Dunnett’s post-hoc test and expressed as mean ± SD (STATISTICA v13 software). Significant differences in comparison with the control were estimated with p<0.05 confidence intervals.

## Results

### Effect of spermidine on expression of genes related to autophagy and epigenetic modifications

Results of relative gene expression in abdomen and head of honey bees supplemented with spermidine at concentrations of 0.1 mM and 1 mM are presented in Table 1 (see also heatmap, S1 Fig). The results showed that both Spd concentrations increased the expression of all analysed genes in the abdomen of honey bees, with the exception of the *DNMT1B* isoform of DNA methyltransferase. In the head, however, only a lower Spd concentration (0.1 mM) caused an increase in the expression of two autophagy-related genes (*ATG9* and *ATG13*), as well as two histone deacetylase isoforms (*HDAC3* and *SIRT1*) and one DNA methyltransferase isoform (*DNMT1A*). There were no significant changes in the expression of the examined genes in honey bee heads supplemented with 1 mM Spd.

**Table 1:** Relative expression in the abdomen and the head of honey bees supplemented with spermidine at 0.1 mM (S0.1) and 1 mM (S1) concentrations for 17-day oral test. The genes examined include: The autophagy-related (ATG) genes and genes of enzymes involved in epigenetic modifications of histones (HDACs—Histone deacetylases, KATs—Histone acetyltransferases) and DNA (DNA methyltransferases).

		*S*_*0*.*1*_				*S*_*1*_			
	*gene*	*Relative expression*	*SE rang*	*p*		*Relative expression*	*SE rang*	*p*	
***Abdomen***	*Autophagy-related genes (ATGs)*								
	ATG3	*2*.*336*	*1*.*673–2*.*683*	*0*.*004*	***up***	*2*.*462*	*2*.*042–3*.*334*	*0*.*001*	***up***
	ATG5	*2*.*926*	*2*.*012–3*.*960*	*0*.*022*	***up***	*3*.*278*	*2*.*574–4*.*191*	*0*.*016*	***up***
	ATG9	*5*.*681*	*3*.*868–9*.*317*	*0*.*012*	***up***	*4*.*378*	*3*.*837–5*.*001*	*0*.*008*	***up***
	ATG13	*3*.*411*	*2*.*854–3*.*987*	*0*.*009*	***up***	*2*.*744*	*2*.*302–3*.*354*	*0*.*006*	***up***
	*Histone deacetylases (HDCAs)*								
	HDAC1	*4*.*145*	*3*.*264–5*.*427*	*0*.*004*	***up***	*4*.*033*	*2*.*739–5*.*647*	*0*.*018*	***up***
	HDAC3	*3*.*659*	*2*.*819–4*.*847*	*0*.*030*	***up***	*2*.*809*	*2*.*007–3*.*866*	*0*.*014*	***up***
	SIRT1	*7*.*646*	*5*.*867–10*.*486*	*0*.*007*	***up***	*5*.*197*	*2*.*970–9*.*940*	*0*.*028*	***up***
	*Histone acetyltransferases (KATs)*								
	P300	*1*.*740*	*1*.*434–2*.*158*	*0*.*028*	***up***	*1*.*577*	*1*.*286–1*.*853*	*0*.*030*	***up***
	KAT6B	*1*.*225*	*1*.*078–1*.*329*	*0*.*004*	***up***	*1*.*224*	*1*.*095–1*.*369*	*0*.*025*	***up***
	KAT2A	*3*.*559*	*2*.*586–5*.*007*	*0*.*019*	***up***	*4*.*696*	*3*.*745–5*.*901*	*0*.*028*	***up***
	*DNA methyltransferases (DNMTs)*								
	DNMT1A	*3*.*048*	*2*.*572–3*.*659*	*0*.*027*	***up***	*4*.*647*	*3*.*603–5*.*975*	*0*.*023*	***up***
	DNMT1B	*1*.*087*	*0*.*912–1*.*302*	*0*.*536*	***-***	*0*.*831*	*0*.*671–0*.*998*	*0*.*115*	***-***
	DNMT3	*2*.*257*	*1*.*360–3*.*151*	*0*.*003*	***up***	*1*.*572*	*1*.*224–2*.*049*	*0*.*015*	***up***
***Head***	*Autophagy-related genes (ATGs)*								
	ATG3	*1*.*189*	*0*.*940–1*.*581*	*0*.*318*	***-***	*1*.*099*	*0*.*779–1*.*478*	*0*.*487*	***-***
	ATG5	*1*.*291*	*0*.*996–1*.*628*	*0*.*121*	***-***	*1*.*094*	*0*.*827–1*.*342*	*0*.*529*	***-***
	ATG9	*1*.*367*	*1*.*109–1*.*1693*	*0*.*040*	***up***	*0*.*967*	*0*.*866–1*.*084*	*0*.*592*	***-***
	ATG13	*1*.*353*	*1*.*035–1*.*731*	*0*.*041*	***up***	*1*.*179*	*0*.*838–1*.*802*	*0*.*410*	***-***
	*Histone deacetylases (HDCAs)*								
	HDAC1	*1*.*455*	*1*.*062–1*.*970*	*0*.*054*	***-***	*0*.*985*	*0*.*681–1*.*375*	*0*.*877*	***-***
	HDAC3	*1*.*299*	*1*.*094–1*.*529*	*0*.*001*	***up***	*1*.*049*	*0*.*718–1*.*511*	*0*.*820*	***-***
	SIRT1	*5*.*008*	*3*.*506–6*.*660*	*0*.*021*	***up***	*1*.*341*	*0*.*821–2*.*017*	*0*.*354*	***-***
	*Histone acetyltransferases (HATs)*								
	P300	*1*.*092*	*0*.*957–1*.*234*	*0*.*263*	***-***	*0*.*928*	*0*.*775–1*.*136*	*0*.*503*	***-***
	KAT6B	*1*.*069*	*0*.*923–1*.*323*	*0*.*597*	***-***	*1*.*090*	*0*.*773–1*.*457*	*0*.*593*	***-***
	KAT2A	*1*.*458*	*1*.*031–2*.*164*	*0*.*121*	***-***	*0*.*972*	*0*.*604–1*.*580*	*0*.*080*	***-***
	*DNA methyltransferases (DNMTs)*								
	DNMT1A	*1*.*513*	*1*.*069–2*.*067*	*0*.*025*	***up***	*1*.*146*	*0*.*663–2*.*048*	*0*.*508*	***-***
	DNMT1B	*1*.*010*	*0*.*873–1*.*224*	*0*.*977*	***-***	*0*.*870*	*0*.*640–1*.*188*	*0*.*422*	***-***
	DNMT3	*1*.*345*	*1*.*093–1*.*711*	*0*.*082*	***-***	*1*.*156*	*0*.*951–1*.*384*	*0*.*220*	***-***

up–means overexpression for p<0.05 confidence intervals significant differences in comparison with control bees.

### Western blot analysis of histone acetylation

**Fig 1:**
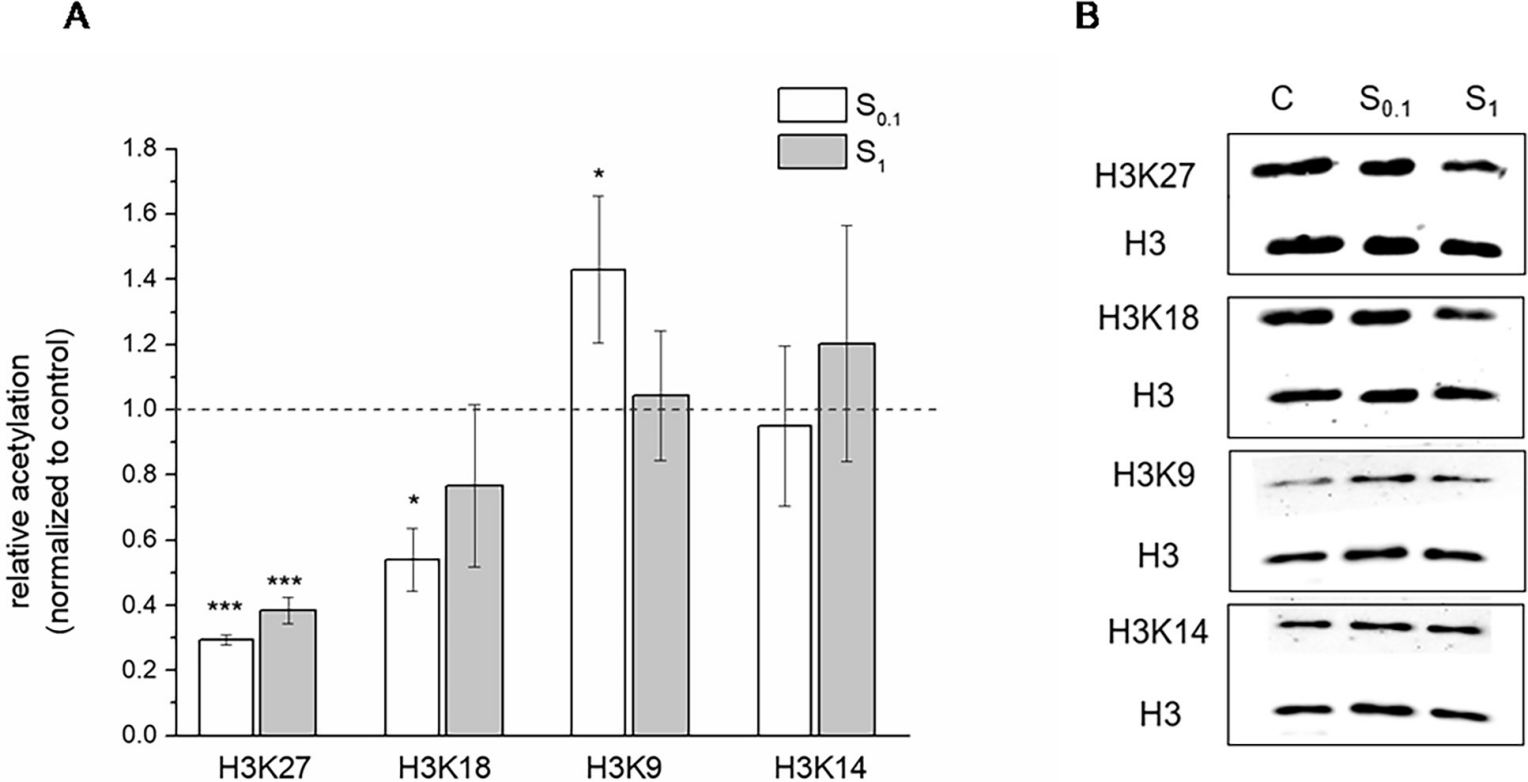
Western blot analysis of histone 3 (H3) acetylation in honey bees supplemented with spermidine at 0.1 mM (S0.1) and 1 mM (S1) concentrations for 17-day oral test. (A) Relative acetylation of H3 (normalized to control considered as 1) on indicated lysine residue determined by quantitative Western blot analysis. Results are shown as mean ± SD (n = 3). Statistical significance in comparison with the control was analysed with Dunnett’s post-hoc test and represented with asterisk (***p<0.001, *p<0.05). (B) Representative blots using site-specific antibodies. Normalization was always done with respect to the band intensity of H3 as a loading control on the same blot. The blots displayed in the figure were cropped; uncropped blots are included in S1 Raw images.

The effect of spermidine supplementation on histone acetylation in honey bees at dosages of 0.1 mM and 1 mM was assessed by Western blot analyses using antibodies that specifically detect acetylation of lysine residues (K9, 14, 18, and 27) at the amino-terminal tail of histone H3 (Fig 1). The results showed hypoacetylation of H3 at position K27 for both tested concentrations, and at position K18 only for the lower concentration of Spd (0.1 mM). On the other hand, hyperacetylation of H3 at K9 for lower Spd concentration and no change in acetylation of H3 at K14 were obtained.

## Discussion

Previously has been shown that spermidine added at millimolar concentrations (particularly 0.1 and 1 mM) to food extended lifespan and improved oxidative status in honey bees [20]. As autophagy one of the more important geroprotective mechanisms of Spd [4,9,11], these findings prompted the present study to examination of the effects of those two Spd concentrations on the expression of *ATG* genes in honey bees. ATG proteins are the core of molecular machinery responsible for sequential activation of autophagy from the initial phagophore to final autolysosome formations [13]. We measured expression of *ATG3*, *5*, *9*, and *13*, involved in early phases of autophagy, and showed that both Spd concentrations, after 17 days, increased expression of all examined genes in the abdomen, but only the lower Spd concentration (0.1 mM) increased expression of *ATG9* and *ATG13* in the head. These results indicate that autophagy was triggered by Spd supplementation and that it was more intense in the abdomen of honey bees. The fact that worker honey bees have major fat body tissue in the abdomen, which is comparable in function to vertebrates’ white adipose tissue and liver [30], could explain increased abdomen sensitivity. It is important to mention that the midgut, a metabolically active tissue, is located in the abdomen as well. The midgut of honey bees is responsible for digestion and nutrient absorption, and in the case of oral supplementation, this is the first gateway for gradients. In regard to that, recorded changes could be attributed to the midgut too. Obtained increased expression of ATGs is in accordance with the study by Eisenberg et al. [9], who obtained up-regulation of *ATG*7, *11*, and *15* in yeast, as well as *ATG8* in human cells, flies, and *C*.*elegances* after Spd treatment. The authors of the same study concluded that autophagy is critical for Spd effects because mutations in some *ATG* genes abrogated Spd-mediated life extension in treated species.

Even though the comprehensive molecular targets of Spd are still incompletely understood, there are indications that some actors are implicated in epigenetic modulations [12,16,18,31,32]. It was suggested that Spd effects are most likely associated with general histone H3 deacetylation that lead to epigenetic reprogramming of the transcriptome and induction of autophagy in yeast and human cells [9,11].

In accordance with the previous statement, the gene expression of selected enzymes involved in histone modifications that could be related to autophagy in supplemented honey bees, such as histone deacetylases (*HDAC*s) and histone acetyltransferases (*KAT*s) were measured. It has been found that gene expression for *HDAC1*, *HDAC3*, and *SIRT1* was up-regulated in the abdomen. Among them, the most interesting is *SIRT1*, which was the most up-regulated not only in the abdomen but also in the head of honey bees. Even though it was shown that Spd stimulates autophagy independently of SIRT1 in yeast and worms [10,33], these findings lead to proposal that SIRT1 may stimulate autophagy in honey bees. Recent studies revealed SIRT1 as a longevity factor and its importance in the regulation of the formation of autophagosomes [34]. SIRT1 is a member of the sirtuin family of NAD^+^-dependent histone/protein deacetylases (HDACs) and acts as a sensor of nutrient availability. SIRT1 can induce autophagy either directly by deacetylating essential autophagic modulators, such as ATG5, ATG7, and ATG8/LC3, or indirectly through a downstream signalling network by deacetylating several transcription factors, such as FoxO proteins, p53, and the p65 component of NF- κB complex [35]. Additional investigations found that Spd induced up-regulation of *SIRT1* and implications for SIRT1-related biological responses in rat and human culture cells, but in those studies, autophagy was not further characterized [16,36,37].

In addition to HDAC activity, KATs also control the histone acetylation landscape. Spd was described as an inhibitor of acetyltransferase activity of the recombinant EP300 protein (E1A-binding protein p300) *in vitro*, and many authors consider that this could be the reason for the H3 hypoacetylation and autophagy induction associated with Spd [9]. The results of the present study showed increased gene expression for *KAT2A*, *KAT6B*, and *P300* in the abdomen but not in the head following Spd treatment. On the other hand, hypoacetylation of H3 at positions K18 and K27, hyperacetylation at K9, and no change in acetylation of K14 was observed. Hypoacetylation at H3K18 and H3K27 is in line with the results of the study by Jin et al. [38], which obtained a reduction of histone acetylation on H3K18 and H3K27 (H3K18/27ac) by deletion of EP300. However, Eisenberg et al. [9] obtained induction of hypoacetylation of H3 at positions K9, 14, and 18 in yeast, as well as at K14 and 18 in human culture cells and hepatocytes from mice fed with Spd. These results confirm the assumed complexity of Spd action on epigenetic and, consequently, autophagy regulation at different levels of gene expression and require additional analyses. Obtained hyperacetylation of H3 at K9 in honey bees could be in relation to up-regulation of *KAT2A*/*2B*, which particularly modified H3 in that position [39].

In the present study, the expression of three DNA methyltransferases in honey bees treated with Spd was also assessed. Up-regulation of *DNMT1A* and *DNMT3* in the abdomen and only *DNMT1A* in the head was observed. DNA methyltransferases are key epigenetic enzymes that regulate gene expression through DNA methylation. Many studies have shown that alterations in DNA methylation status have a significant impact on longevity and age-related diseases [30]. Mostly, there is a genome-wide decline in DNA methylation with aging [40]. Recent studies indicated that changes in polyamine metabolism affect the concentrations of substances and enzyme activities involved in DNA methylation due to relationship between polyamine metabolism and DNA methylation (reviewed in [31]). The results of present study are in line with this observation. It has been shown before that Spd supplementation prolonged lifespan and increased endogenous Spd levels in honey bees [20], and here was demonstrated up-regulation of *DNMT* genes. On the other hand, Cardoso-Júnior et al. [41] showed that *DNMTs* expression is positively associated with age in the abdomen of honey bees. More accurately, they measured that *DNMT1A* and *DNMT3* expressions were up-regulated in the abdomens, whereas *DNMT1B* and *DNMT2* were down-regulated in the heads of old workers. Further, they showed that treatment of newly emerged honey bee workers with the DNMT inhibitor (RG108) increased lifespan, and probably genomic DNA methylation status, indirectly affecting the expression of vitellogenin as an aging-related gene. These results seem to be in contrast with the results of present study and with the general opinion that aging is associated with global DNA hypomethylation and site-specific hypermethylation [42,43]; however, the mechanisms underlying age-related DNA methylation changes remain mostly undiscovered [44].

In conclusion, the findings of present study show that supplementing spermidine at levels favourable to increasing honey bee lifespan generates profound epigenetic changes and promotes expression of autophagy related genes. It should be also noted that the findings in this study have a limited broad due to the use of workers with similar genomes from the single colony. More research is needed to determine the precise mechanism of spermidine activity and to establish a clearer link between epigenetic alterations and consequently to autophagy induction in honey bees. Nonetheless, these findings show that spermidine most likely triggers autophagy, which may be an important geroprotective mechanism in honey bees as well.

## Supporting information

S1 Fig Heatmap illustrating gene expressions in honey bees supplemented with spermidine. (DOCX)

S1 Table List of primers used for quantitative PCR. (DOC)

S2 Table List of antibodies with dilutions used for Western blot analysis. (DOCX)

S1 Raw images Uncropped blots used in Fig 1. (PDF)
