# Supplementary material for: Spermidine supplementation in honey bees: Autophagy and epigenetic modifications
Source: PLoS One. 2024 Jul 1;19(7):e0306430. doi: 10.1371/journal.pone.0306430 (PMC11216588; doi:10.1371/journal.pone.0306430)
Supplement: S1 Fig — (DOCX) [file pone.0306430.s001.docx]

**S1 Figure**


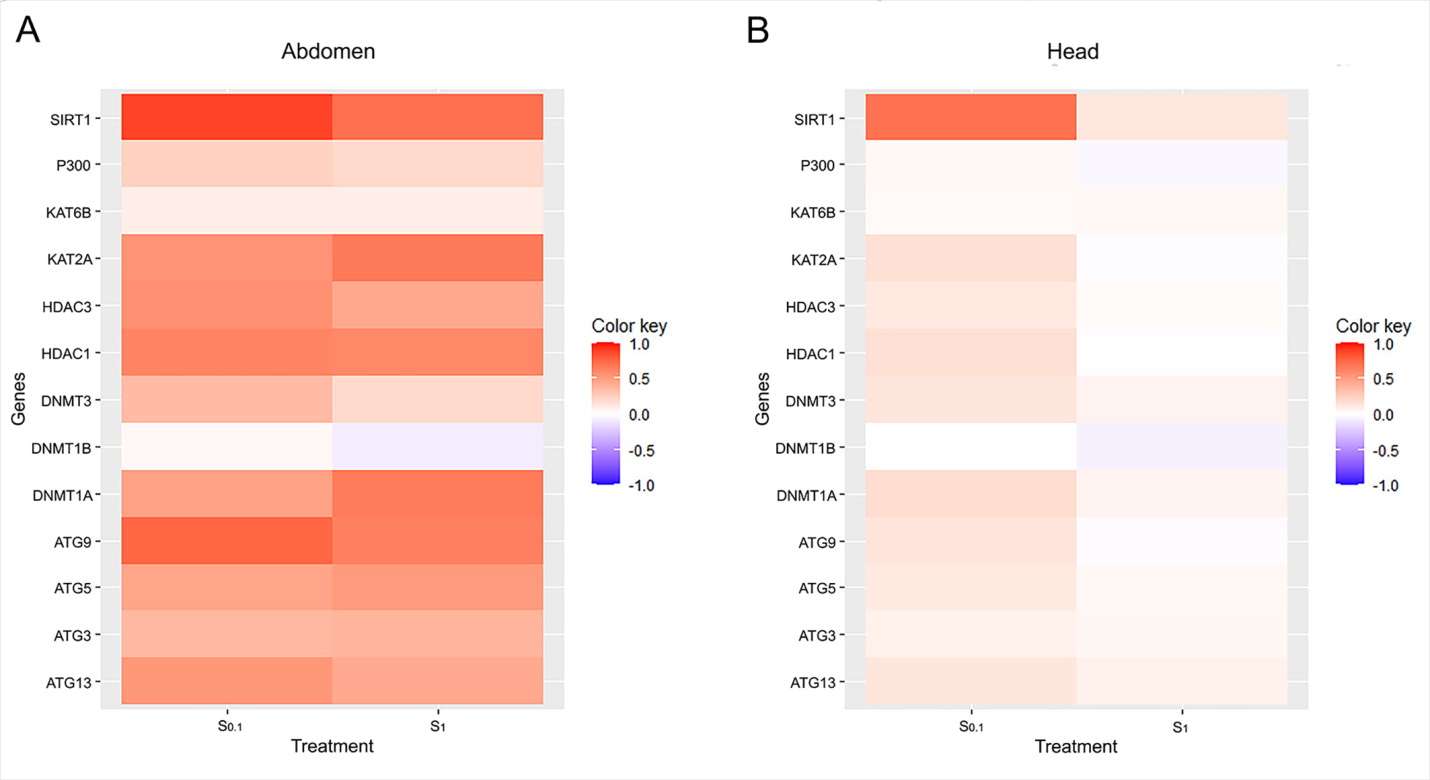


S1 Figure. Heatmap illustrating gene expressions in A) abdomen and B) head of honey bees supplemented with spermidine at 0.1 mM (S0.1) and 1 mM (S1) concentrations after a 17-day oral test in comparison with control bees. Examined genes included: autophagy-related (*ATG*) genes and genes for enzymes involved in epigenetic modifications of histones (*HDAC*s-Histone deacetylases and *KAT*s- Histone acetyltransferases) and DNA (*DNMT*s- DNA methyltransferases). The color key showing the log2 expression values is shown to the right of the panel.
