# Supplementary material for: Spermidine supplementation in honey bees: Autophagy and epigenetic modifications
Source: PLoS One. 2024 Jul 1;19(7):e0306430. doi: 10.1371/journal.pone.0306430 (PMC11216588; doi:10.1371/journal.pone.0306430)
Supplement: S1 Table — (DOC) [file pone.0306430.s002.doc]

Table S1. List of primers used for quantitative PCR.

| **Amplification target** | **Sequence** | **Efficiency** | **Reference** |
| --- | --- | --- | --- |
| β-actin (reference gene) (**ActB**) | F: ATGCCAACACTGTCCTTTCTGG  R: GACCCACCAATCCATACGGA | 105% | [1] |
| Ribosomal protein 49  (reference gene) (**Rp49**) | F: CGTCATATGTTGCCAACTGGT  R: TTGAGCACGTTCAACAATGG | 88% | [2] |
| Autophagy-related protein 9 (**ATG9**) | F: TCGCCAAAAACGAAGATGCTTGTG  R: TTGCTCGTCGGTTTGGCTCT | 90% | NCBI PrimerBlast* |
| Autophagy-related protein 5 (ATG5) | F: TGCCGTTGTATTCATAATGGCGA  R: CACAAATTTCCTCAGAATCAAGCGT | 89% | NCBI PrimerBlast* |
| Autophagy-related protein 3 (**ATG3**) | F: GGTACTGCGCTTGGTGTTGC  R: AGCCCATTGCCATGTTGGACA | 93 % | NCBI PrimerBlast* |
| Autophagy-related protein 13 (**ATG13**) | F: GTAGATTTGAAAACTCCTTTCGCCG  R: GCGAGCGTTCTTTCCTCCATAA | 95% | NCBI PrimerBlast* |
| Histone deacetylase 1 (rpd3)(**HDAC1**) | F: GGTTCTGTAGCTGCTGCGGTGA  R: GGCATGATGTAAACCACCACCCC | 104% | [3] |
| NAD-dependent protein deacetylase sirtuin-1 (132 bp) (**SIRT1**) | F: GCTCCTTTGCTACTGCATCG  R: CCGAAATGGGAGGCAAAGTG | 99% | NCBI PrimerBlast* |
| Histone deacetylase 3 (132 bp) (**HDAC3**) | F: TTGGAGCAGAAAGTGGGAGA  R: CTATTGCTGTCGGTTGGAAAA | 108% | [4] |
| Histone acetyltransferase p300 (102 bp) (**P300**) | F: CCCTTCTCGGTTATGGTGGC  R: GGAGGTCCCATCACACCTTG | 92% | NCBI PrimerBlast* |
| Histone acetyltransferase KAT6B (130 bp) (**KAT6B**) | F: CGAACGAAAAGCGAGGCACG  R: ACACCGCGCTCACAGTTACG | 98% | NCBI PrimerBlast* |
| Histone acetyltransferase KAT2A (103 bp) (**KAT2A**) | F: GGACCAGAACCAATGGAAGC  R: ATGGCCATGCTGTACTGTGG | 107% | NCBI PrimerBlast* |
| DNA methyltransferase 1A (118 bp) (**DNMT1A**) | F: CGAGTAGTAAGCGTGCGTGAA  R: CAAGTGGTGGAGGAACTGC | 95% | [5] |
| DNA methyltransferase 1B (219 bp) (**DNMT1B**) | F: GAAATTACATGGGTGGGAGAA  R: GTCACTGCCTCTTCGAAACC | 88% | [6] |
| DNA methyltransferase 3 (103 bp) (**DNMT3**) | F: GGATGGAGAAAATGCGCGAC  R: TCGAATCCTAGACGGGAGCA | 92% | NCBI PrimerBlast* |

*Primers designed by using the NCBI PrimerBlast [7].

**Supporting References**

1. Antúnez K, Martín-Hernández R, Prieto L, Meana A, Zunino P, Higes M. 2009 Immune suppression in the honey bee (*Apis mellifera*) following infection by Nosema ceranae (Microsporidia). *Environ. Microbiol.* **11**, 2284–90. (doi:10.1111/j.1462-2920.2009.01953.x)

2. Lourenço AP, Mackert A, dos Santos Cristino A, Simões ZLP. 2008 Validation of reference genes for gene expression studies in the honey bee, *Apis mellifera*, by quantitative real-time RT-PCR. *Apidologie* **39**, 372–385. (doi:10.1051/apido:2008015)

3. Wang H, Zhang S-W, Zeng Z-J, Yan W-Y. 2014 Nutrition affects longevity and gene expression in honey bee (*Apis mellifera*) workers. *Apidologie* **45**, 618–625. (doi:10.1007/s13592-014-0276-3)

4. Wang WX, Tian LQ, Huang Q, Wu XB, Zeng ZJ. 2014 Effects of 10-Hydroxy-2-decenoic acid on the development of honey bee (*Apis mellifera*) larvae. *J. Apic. Res.* **53**, 171–176. (doi:10.3896/IBRA.1.53.1.19)

5. Cardoso-Júnior CAM, Guidugli-Lazzarini KR, Hartfelder K. 2018 DNA methylation affects the lifespan of honey bee (*Apis mellifera* L.) workers – Evidence for a regulatory module that involves vitellogenin expression but is independent of juvenile hormone function. *Insect Biochem. Mol. Biol.* **92**, 21–29. (doi:10.1016/j.ibmb.2017.11.005)

6. Biergans SD, Giovanni Galizia C, Reinhard J, Claudianos C. 2015 Dnmts and Tet target memory-associated genes after appetitive olfactory training in honey bees. *Sci. Rep.* **5**, 16223. (doi:10.1038/srep16223)

7. Ye J, Coulouris G, Zaretskaya I, Cutcutache I, Rozen S, Madden TL. 2012 Primer-BLAST: A tool to design target-specific primers for polymerase chain reaction. *BMC Bioinformatics* **13**, 134. (doi:10.1186/1471-2105-13-134)
