# Supplementary material for: Spermidine supplementation in honey bees: Autophagy and epigenetic modifications
Source: PLoS One. 2024 Jul 1;19(7):e0306430. doi: 10.1371/journal.pone.0306430 (PMC11216588; doi:10.1371/journal.pone.0306430)
Supplement: S2 Table — (DOCX) [file pone.0306430.s003.docx]

Table S2. List of antibodies with dilutions used for Western blot analysis.

| Antibody | Type* | Dilution** | Suplier |
| --- | --- | --- | --- |
| Anti-Histone H3 - Nuclear Loading Control (ab1791) | RP | 1:80000 | Abcam |
| Anti-Histone H3 (acetyl K27) (ab4729) | RP | 1:5000 | Abcam |
| Anti-Histone H3 (acetyl K9) (ab4441) | RP | 1:10000 | Abcam |
| Anti-Histone H3 (acetyl K18) (ab1191) | RP | 1:1000 | Abcam |
| Anti-Histone H3 (acetyl K14) (ab52946) | RM | 1:2000 | Abcam |
| Recombinant Anti-histone H3 (acetyl K4) (ab176799) | RM | 1:10000 | Abcam |
| Recombinant Anti-histone H3 (acetyl K23) (ab177275) | RM | 1:1000 | Abcam |
| Anti-rabbit IGG-Peroxidase antibody produced in goat- secondary antibody(A9169) | GP | 1:80000 | Sigma-Aldrich |

*Abbreviations: RP - Rabbit polyclonal; RM-Rabbit monoclonal, GP-Goat polyclonal

**Antibodies were prepared (diluted) in 3% non-fat dried milk in TBST
