# Supplementary material for: Spermidine supplementation in honey bees: Autophagy and epigenetic modifications
Source: PLoS One. 2024 Jul 1;19(7):e0306430. doi: 10.1371/journal.pone.0306430 (PMC11216588; doi:10.1371/journal.pone.0306430)

Uncropped blots used in Fig 1

For all bolts, the ELC method was used, and because of that, protein markers (prestained protein markers) are not visible.

Blot 1

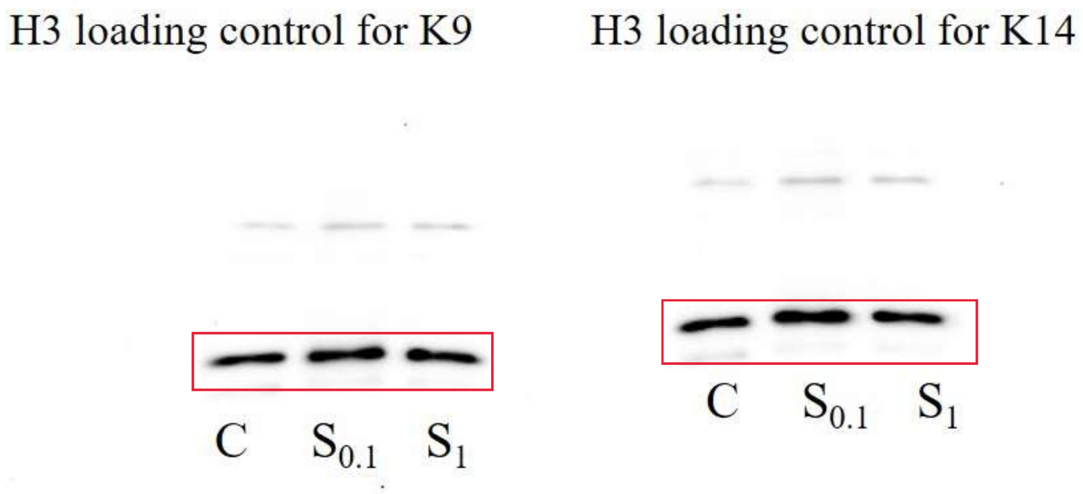

Blot 2

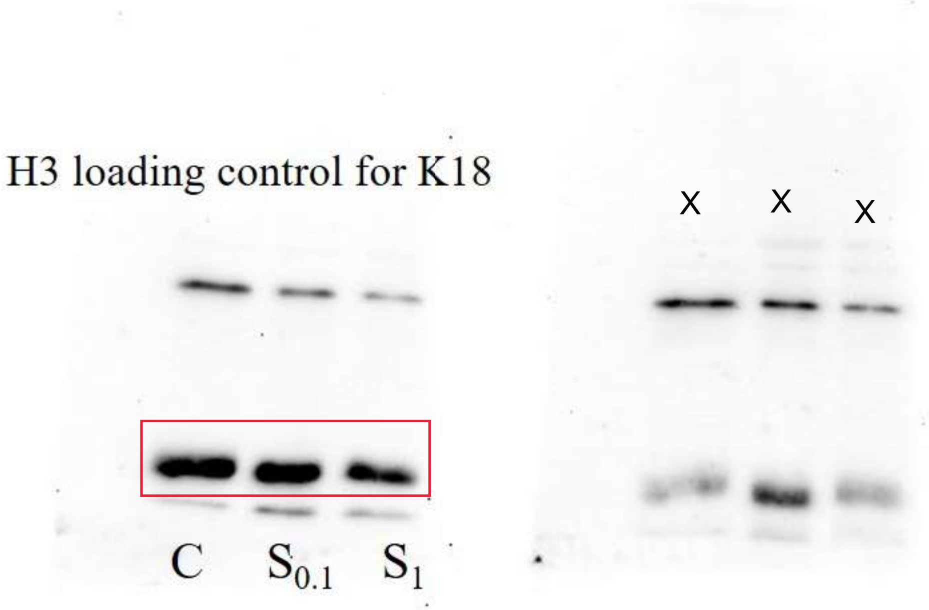

Blot 3

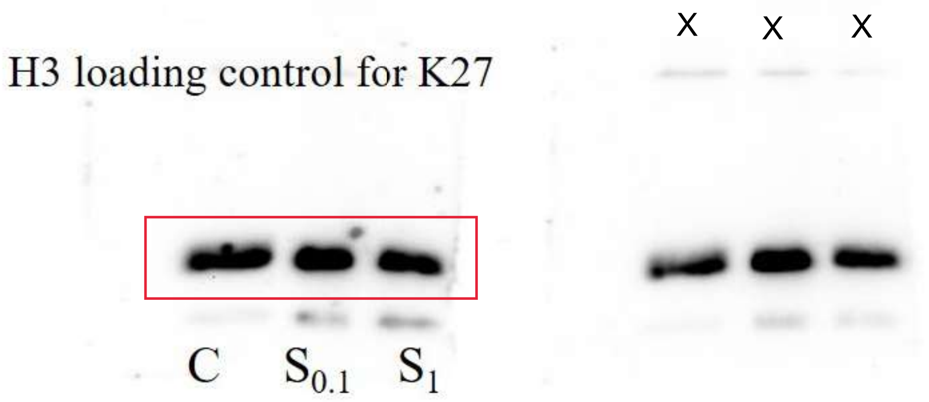

Blot 4

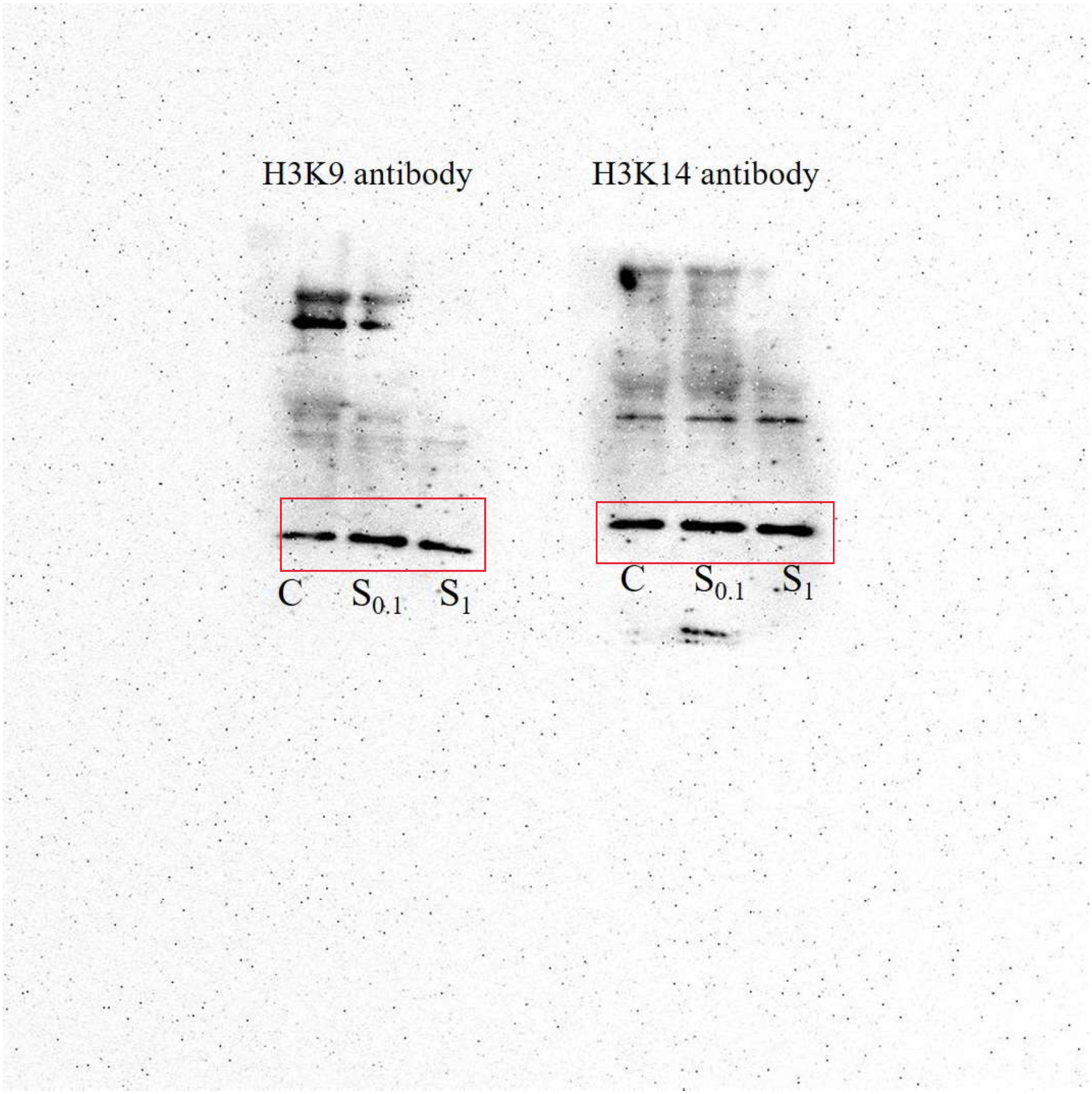

Blot 5

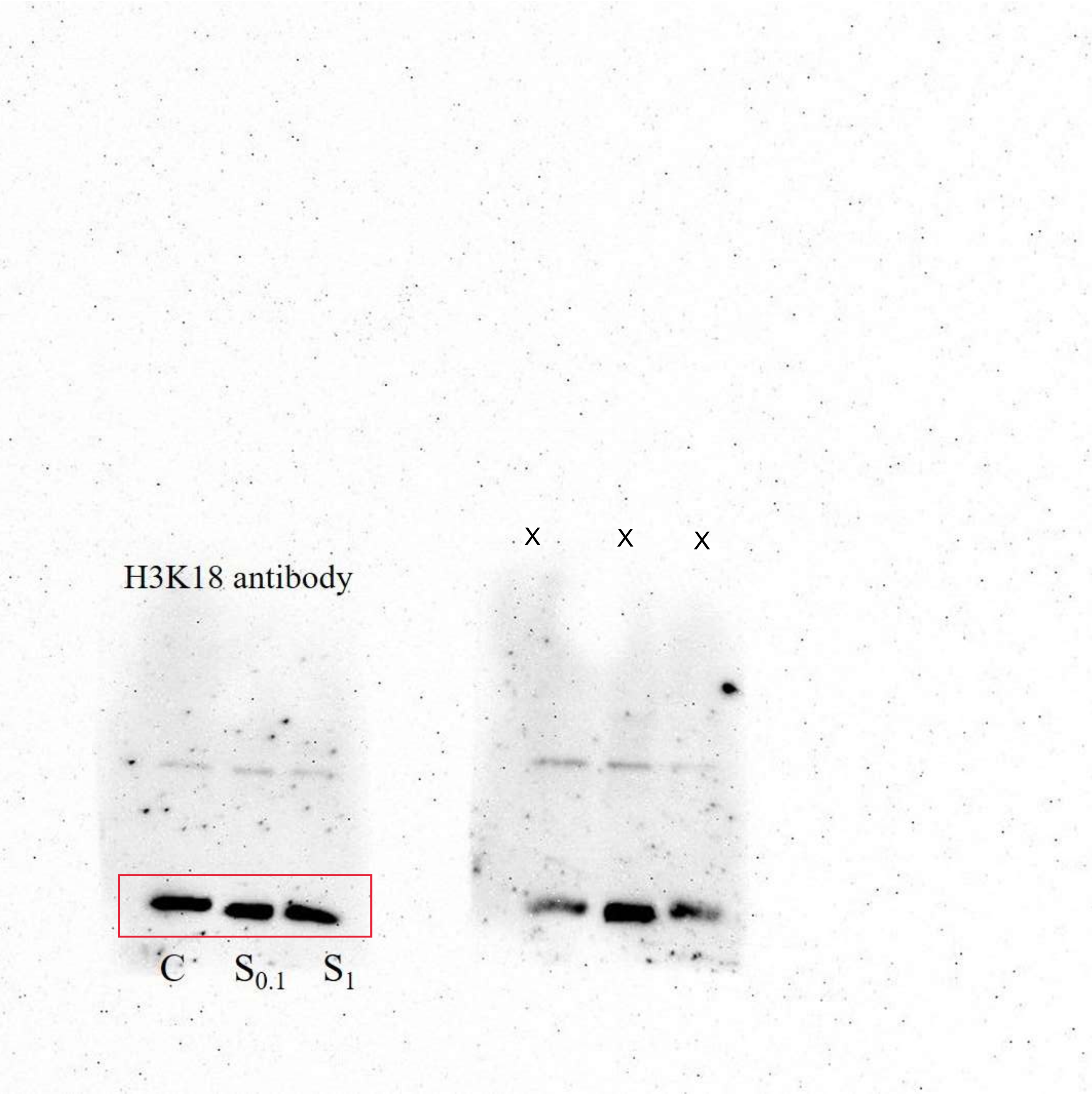

Blot 6

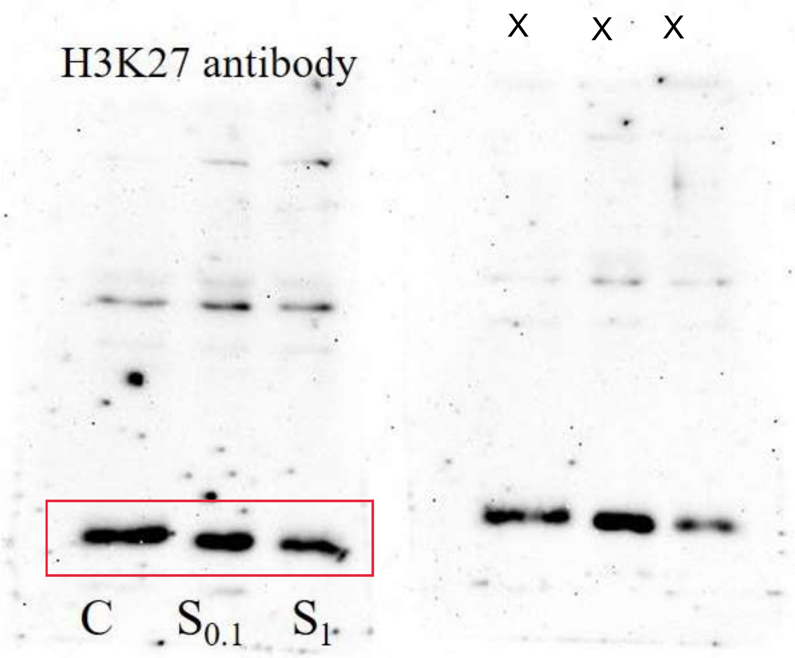

Supplement: S1 Raw images — (PDF) [file pone.0306430.s004.pdf]
